# Angiogenesis Is Differentially Modulated by Platelet-Derived Products

**DOI:** 10.3390/biomedicines9030251

**Published:** 2021-03-04

**Authors:** Sarah Berndt, Gilles Carpentier, Antoine Turzi, Frédéric Borlat, Muriel Cuendet, Ali Modarressi

**Affiliations:** 1 Department of Plastic, Reconstructive and Aesthetic Surgery, Geneva University Hospitals, Faculty of Medicine, 1205 Geneva, Switzerland; Ali.Modarressi@hcuge.ch; 2 Faculty of Medicine, Geneva University, 1205 Geneva, Switzerland; 3 Regen Lab SA, 1052 Le Mont-sur-Lausanne, Switzerland; aturzi@regenlab.com; 4 Gly-CRRET Research Unit 4397, Paris-Est Créteil University, 94000 Créteil, France; image.bio.methods@free.fr; 5 School of Pharmaceutical Sciences, University of Geneva, 1205 Geneva, Switzerland; fborlat@unige.ch (F.B.); mcuendet@unige.ch (M.C.); 6 Institute of Pharmaceutical Sciences of Western Switzerland, University of Geneva, 1205 Geneva, Switzerland

**Keywords:** angiogenesis, platelet-rich-plasma, endothelial cells, secretome

## Abstract

Platelet-derived preparations are being used in clinic for their role in tissue repair and regenerative processes. The release of platelet-derived products such as autologous growth factors, cytokines and chemokines can trigger therapeutic angiogenesis. In this in vitro study, we evaluated and compared the ability of three platelet-derived preparations: platelet-rich-plasma (PRP), PRP-hyaluronic acid (PRP-HA) and platelet lysates (PL) at various concentrations (5–40%) to modulate human umbilical vein endothelial cells (HUVEC) biological effects on metabolism, viability, senescence, angiogenic factors secretion and angiogenic capacities in 2D (endothelial tube formation assay or EFTA) and in 3D (fibrin bead assay or FBA). HUVEC exocytosis was stimulated with PRP and PRP-HA. Cell viability was strongly increased by PRP and PRP-HA but mildly by PL. The three preparations inhibit HUVEC tube formation on Matrigel, while PRP enhanced the complexity of the network. In the fibrin bead assay (FBA), PRP and PRP-HA stimulated all steps of the angiogenic process resulting in massive sprouting of a branched microvessel network, while PL showed a weaker angiogenic response. Secretome profiling revealed modulation of 26 human angiogenic proteins upon treatment with the platelet derived preparations. These in vitro experiments suggest that PRP and PRP-HA are effective biological therapeutic tools when sustained therapeutic angiogenesis is needed.

## 1. Introduction

Wound repair is a complex physiological process for the regeneration of damaged tissues, and involves interplay between numerous cell types, cytokines, healing mediators and the vascular system [1].

Chronic wounds are generally referred to as wounds that fail to heal through the body’s natural healing process. Despite the extensive research to improve wound healing management and the wide range of newly developed products, the management of chronic and full-thickness wounds is still a big challenge. The major obstacles in the process of healing include prolonged infection, continuous inflammation, and particularly, impaired healing processes such as a lack of angiogenesis, epithelial migration, and cell proliferation [2]. New techniques in tissue regeneration include biological tissue replacement, gene therapy, recombinant growth factors (GF) and cell-based treatments [3].

Autologous blood derived-products like platelet-rich plasma (PRP) are alternative therapies for the promotion of tissue repair and angiogenesis. For more than ten years, the regenerative potential of PRP has been demonstrated in a range of tissue types including skin, bone, cartilage, tendon and muscle and to improve tissue healing after surgery [4].

PRP, as the fraction of blood plasma with concentrated platelets, has a reservoir of growth factors present in both the plasma and the platelets. It has been estimated that platelets contain over 300 biologically active factors that can be released in situ upon activation. Cytokines released from activated platelets can impact the local environment, supporting angiogenesis, blood vessel growth, and tumor growth [5]. In the human body, platelets play a critical role in regulating new blood vessel formation through a wide range of activators and inhibitors of angiogenesis and by several pathways, including differential exocytosis of angiogenesis regulators stored in their alpha granules [6]. Nevertheless, their contribution to blood vessel repair in the course of wound healing is still poorly understood [7].

PRP may have different physical forms (from liquid to gel) depending on the presence of additives such as anticoagulants or procoagulants, that inhibit or activate the coagulation process ex vivo leading to the formation of a platelet-rich fibrin network. PRP can also be used in combination with hyaluronic acid (HA), to form a platelet-enriched hydrogel. Other blood-derived products include human serum and human platelet lysates (PL) which are obtained following lysis of the platelets and collection of the released components. Evaluating the effect of crucial angiogenic factors released from different platelet preparations could thus be useful in tissue regeneration and wound healing. This would be informative for physician to guide their product selection as many different medical devices to prepare PRP are accessible [8].

In this work, we focused on three platelet-based products: freshly prepared PRP, a combination of PRP and HA and PL. They differ in terms of their GF, cytokine and chemokine composition due to different preparation protocols. PL contains more GF than the other preparations because the GF are extracted from the platelets by heavy centrifugation that disrupts platelet cellular membranes [9].

For PRP preparation, we have used the CuteCell^TM^ PRP medical device, that allowed the preparation of autologous platelet-rich plasma to be used as culture media supplement for in-vitro autologous cells expansion in GMP conditions. CuteCell^TM^ PRP tubes are designed for rapid preparation of platelet rich plasma from a small volume of the patient’s blood in a closed-circuit system. Blood is drawn from the patient directly in the evacuated tubes containing sodium citrate as anticoagulant and then spun in a centrifuge accordingly to centrifuge operating instructions. At the end of the centrifugation, blood components are separated thanks to the thyxotropic separation gel and the platelet-rich plasma can easily be collected from the device.

For the preparation of PRP-HA combination, we have developed a specific medical device called “Cellular Matrix^TM^”. It is the same technology as the CuteCell^TM^ PRP device except that the device contains 2 mL of natural, non-crosslinked-HA (1550 kDa) at a concentration of 20 mg/mL.

HA is one of the main component of the extracellular matrix. It is considered as a key player in the tissue regeneration process. It has been recognized as a potent modulator of the main phases of wound healing via specific HA receptors, and more specifically on angiogenesis [10,11]. Studies have shown that the combination of PRP and HA are more effective for the treatment of moderate knee osteoarthritis than the use of HA or PRP alone [12]. PRP and HA combination treatment significantly improved arthralgia, reduced humoral and cellular immune responses and promoted angiogenesis, which improved the patients’ histological parameters compared with PRP or HA treatment alone [13]. Moreover, an in vitro study showed that hyaluronic acid hydrogel increases the GF release by PRP after five days [14]. We thus thought that testing those synergistic effects in in vitro angiogenesis would be interesting.

The PL tested in this work was a ready-to-use commercial preparation of activated growth factors, that has a longer shelf life upon cold storage −80 °C [9,15], while PRP and PRP-HA were freshly prepared and the platelets were not activated prior to use [16,17].

The formation of vascular networks during the angiogenic process relies on the association of cells into stable 3D tubes through differentiation, migration, proliferation, aggregation, and rearrangement of these cells to form cords that then undergo lumen formation. To model vascular morphogenesis, we used simple in vitro assays (2D endothelial cell cultures) and complex 3D models where a matrix is incorporated to more closely mimic the native in vivo environment.

## 2. Materials and Methods

### 2.1. Preparation of Autologous PRP, PRP-HA and PL

Blood samples were obtained from healthy donors. The procedure conformed to the principles of the Declaration of Helsinki and was approved by CCER Geneva ethics committee (ID 2017-00700, approved on 18 October 2018).

For each donor, 30 mL of human peripheral blood was collected into two specific medical devices: two CuteCell^TM^ PRP and one CellularMatrix^TM^ ACP-HA (Regen Lab SA, Le Mont-sur Lausanne, Switzerland). The collected blood was centrifuged for 5 min in a standard laboratory centrifuge at 1500× *g* at room temperature. Subsequently, the red and white blood cells accumulated at the bottom of the tube under the separator gel, whereas the plasma and platelets remained above the gel layer. Plasma containing platelets was homogenized by inverting the tube five times. The resulting 6 mL of PRP per CuteCell^TM^ PRP tube and 3 mL of PRP for 2 mL of HA per Cellular Matrix^TM^ tube was transferred in a polypropylene tube (Becton-Dickinson, Franklin Lakes, NJ, USA) until use. Platelets, red and white blood cells concentrations, as well as MPV (mean platelet volume) in whole blood in the prepared PRP before addition to culture media were in the same range as previously described [17]. PL (commercial name PLTmax) was prepared according to the manufacturer’s instructions (Merck, Darmstadt, Germany).

### 2.2. Primary Cell Culture

HUVECs (human vascular endothelial cells) (PromoCell, Heidelberg, Germany) were cultured in EBM-2 (endothelial basal medium) (PromoCell) supplemented with the Supplement Mix (PromoCell) that contains 2% fetal bovine serum, thus constituting ECGM-2. The Supplement Mix consists of 5 ng/mL epidermal growth factor, 10 mg/mL basic fibroblast growth factor, 20 ng/mL insulin-like growth factor, 0.5 ng/mL vascular endothelial growth factor 165, 1 µg/mL ascorbic acid, 22.5 µg/mL heparin and 0.2 µg/mL hydrocortisone. In some experiments, cells were cultured in EBM-2 with the supplement mix but without 2% FBS. In all experiments, cells between passages 2 and 7 were used. NHDFs (normal human dermal fibroblasts) from juvenile foreskin (PromoCell) were grown in complete FGM (fibroblast growth medium) (PromoCell) supplemented with 5 µg/mL insulin and 1 ng/mL human basic fibroblast growth factor (PromoCell). NHDFs were used between passages 2 and 9. All cells were cultured in 5% CO_2_ at 37 °C, and media were replaced every 2 days.

### 2.3. Cell Growth, Metabolic Activity, Proliferation

#### 2.3.1. MTT Cell Growth Assay

HUVECs were seeded in 96-well plates at a density of 1 × 104 cells per well and incubated overnight in EBM-2 medium containing 0.5% FBS (Fetal bovine Serum). The following day, cell cultures were treated for 72 h with a range of PRP, PRP-HA or PL concentrations (5–40%) and then 10 µL MTT was added (final concentration 500 µg/mL) for 4 h at 37 °C. Images were taken under an inverted microscope (10×). The medium was then aspirated, and insoluble formazan crystals were dissolved with 100 µL DMSO per well. Absorbance was read at 595 nm on a microplate reader (Biotek, Winooski, VT, USA)

#### 2.3.2. Crystal Violet Viability Assay

HUVEC were plated at a density of 1 × 104 cells per well in 96-well plates and treated for 72 h with heparin (5I U/mL), PRP (5–40%), PRP-HA (5–40%), PL (5–40%). Plates were washed three times with PBS (phosphate buffered saline), fixed with 4% paraformaldehyde for 10 min, stained with 0.1% crystal violet, and samples were rinsed in running water and air-dried. Images were taken under an Eclipse Ts2-FL inverted microscope fitted with a DS-Fi3 camera and DS-L4 control unit (Nikon corporation, Tokyo, Japan). Samples were dissolved with glacial acetic acid, and the optical density at 570 nm was determined using an automatic microplate reader (Biotek).

### 2.4. Endothelial Tube Formation Assay (ETFA)

Endothelial network formation in the ETFA was carried out by seeding HUVEC (105 cells/well) on Matrigel (200 µL/well) into a 24-well plate for 6 h at 37 °C with 5% CO_2_. Cells were suspended in EGM-2 (endothelial growth medium), heparin (control condition), or with 10% PRP, 10% PRP-HA or 10% PL. Five pictures per well (center of the well and four cardinal points) were taken after 6h using a 10× objective in phase contrast mode after 4% paraformaldehyde fixation. For statistical analyses, four to six wells were seeded per condition. The image analysis procedure can be found here [18].

### 2.5. Fibrin Bead Assay (FBA)

The FBA was performed by adhering HUVEC to dextran coated Cytodex 3 microcarriers beads embedded in a fibrin gel as described in [18]. Dry Cytodex 3 microcarrier beads were hydrated in PBS for at least 3 h at RT and autoclaved for 15 min at 115 °C. The culture medium used in the assay was EGM-2. HUVEC were mixed with Cytodex 3 microcarrier beads at a cell density of 400 cells per bead in a solution of 2500 beads per ml of EGM-2 medium. Beads and HUVEC were then co-incubated in a humidified incubator at 37 °C and 5% CO_2_ and gently shaken manually every 20 min for 4 h to allow cell adherence to the bead surface. Beads with adherent cells were transferred to a 75 cm^2^ tissue culture flask and were incubated for a further 24 h. Cells coated on beads were then washed three times with 1 mL of EGM-2 to remove non-coated cells and were resuspended at a density of 1000 beads/mL in a solution of fibrinogen type I (2.5 mg/mL) with 0.15 U/mL of aprotinin at pH 7.4. To polymerize the fibrin in the wells, 4 µL of thrombin (10 units/mL) were deposited into each well of a 96-well-optical plate (BD Falcon) suitable for high throughput imagers. Then, 80 µL of the fibrinogen type I-aprotinin-HUVEC coated bead solution were deposited over the thrombin drops, gently mixed by slowly pipetting up and down and allowed to clot for 2 min at room temperature. The plate was then placed at 37 °C and 5% CO_2_ for 30 min to allow gel formation. Eighty µL of EGM-2 medium was added to each well and allowed to equilibrate with the bead-containing gels for 30 min at 37 °C and 5% CO_2_. Following incubation, 3700 NHDF were added and allowed to adhere to the top of the gel. After 1 h, EGM-2 was replaced with fresh medium with or without the 3 platelet-derived preparations. Medium was changed one day later and then every two days. Sprouting was apparent between days 2 and 3, and cultures were imaged at day 4. To quantify the sprouting pseudo-microvessel network, samples were automatically scanned with a high-throughput imager (IXM, Molecular Devices, San Jose, CA, USA) at a 4× magnification with a definition of 4 quadrants (images) per well to cover the entire surface of each well. Eight wells were analyzed per condition. The image analysis procedure can be found here [18].

### 2.6. Image Analysis

Image acquisition was done manually on an inverted microscope for ETFA, and automatically with a high throughput imager for FBA. Image analysis for the FBA and ETFA was performed using a program developed for the ImageJ software [19] on both models. This plugin is an extension of the “Angiogenesis Analyzer” for ImageJ written in the macro language of ImageJ as described previously [18,20].

### 2.7. Angiogenesis Protein Array

RayBio Human Angiogenesis Antibody Arrays C-1000 (RayBiotech, Peachtree Corners, GA, USA) were used to assay the angiogenic factors secreted in conditioned medium containing 10% PRP, 10% PRP-HA or 10% PL as supplements in the presence or in the absence of HUVEC. To prepare the conditioned medium, HUVECs were seeded in T25 flasks at a concentration of 5 × 105 cells per flask in ECGM-2. The following day, the medium was replaced with EBM-2 and 0.5% FBS, and the cells were treated with heparin 2IU/mL as control, 10% PRP, 10% PRP-HA or 10% PL. After 48 h, the medium was collected and centrifuged at 400× *g* for 5 min to remove cellular debris. The supernatants were collected and frozen at −80 °C until further use. 10% PRP, 10% PRP-HA or 10% PL incubated for 48 h alone, were collected at the same time.

Total protein concentration was determined by the Lowry protein assay. In the array, 43 angiogenic cytokines, chemokines, enzymes and growth factors were measured according to the manufacturer’s protocol. Membranes were imaged using a Fusion System (Vilber Lourmat, Marne-la-Vallée, France). Protein levels were quantified using the Protein Array Analyzer in the ImageJ program [21]. Values were normalized to reference spots on the membranes.

### 2.8. HUVEC Senescence Assay

The cells were first examined under a light microscope (Eclipse, Nikon) at 10× objective to monitor morphological changes. Senescent cells present a different morphology (enlarged and flat), unlike proliferating cells, which show a spindle-shaped morphology. β-galactosidase activity is used to identify cellular aging. Cellular aging leads to an increase of intracellular β-galactosidase, an enzyme that degrades the X-galactosidase (X-gal) substrate under a pH value of 6.0, resulting in an increase of a blue product [22]. Thus, β-galactosidase activity can be detected by histochemical staining.

HUVEC were cultured from passage 2 to 6 in the presence of the following treatments: 10% FBS, heparin 2UI/mL, 10% PRP, 10% PRP-HA and 10% PL at a low plating density (2000 cells/cm^2^). At passage 6, cells were cultured in six-well plates to achieve 60–80% confluence, and were then rinsed twice with PBS and fixed with 4% paraformaldehyde and 0.2% glutaraldehyde at 4 °C for 5 min. Subsequently, the fixing solutions were discarded, and the cells were washed with PBS thrice for 3 min. After the PBS was discarded, 1 mL X-gal staining solution containing 20 mmol/L X-gal, 40 mmol/L acetic acid-sodium phosphate (pH 6.0), 5 mmol/L potassium ferrocyanide, 150 mmol/L NaCl, 2 mmol/L magnesium chloride were added to each well according to the instructions of the SA-β-galactosidase staining kit (Cell Signaling Technology, MA, USA). The cells were then incubated at 37 °C overnight and some of the cells became blue. The cells were quantified under an optical microscope (Eclipse, Nikon). The blue-stained positive cells among four pictures (10× objective) per well were observed and counted to obtain the percentage of positive cells, which represented the percentage of senescent cells.

### 2.9. Statistical Analysis

The data are expressed as means +/− standard deviation (SD) One-way analysis of variance (ANOVA) followed by Tukey’s multiple-comparison tests (GraphPad Prism) was made for multiple comparisons analyses. Differences with *p* values of <0.05 were considered to be statistically significant.

## 3. Results

### 3.1. Effect of Platelet-Derived Products on HUVEC Cell Behavior

**Figure 1:**
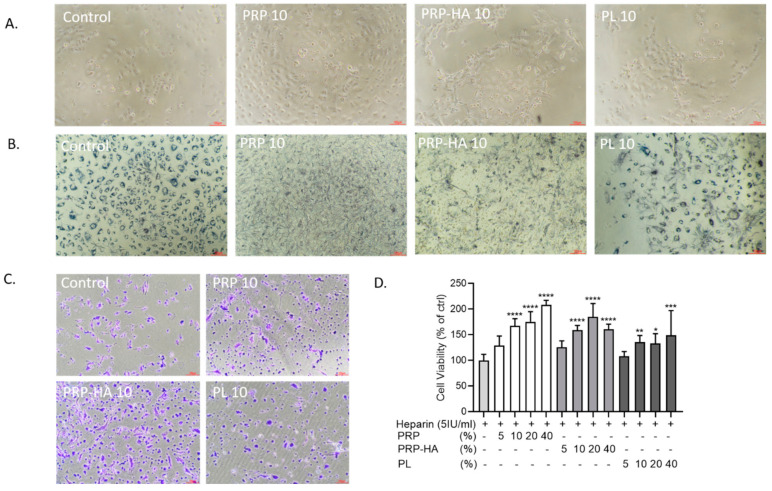
Effect of platelet-derived products on HUVEC cell behavior. (**A**). Bright-field optical photography of HUVEC in the presence of heparin, 10% PRP, 10% PRP-HA or PL after 3 days of culture (**B**). Cellular localization of the MTT formazan in HUVEC incubated for 72 h in different platelet-derived products. HUVEC in control medium and in PL have intracytoplasmic dark granules, while PRP and PRP-HA treated HUVEC show extruded formazan crystals. (**C**). HUVEC viability assessed by crystal violet staining in control and in PRP-, PRP-HA- and PL-treated cultures. (**D**). Quantification of the amount of the released dye by absorbance measurement (590 nm). N = 8, * *p* < 0.05, ** *p* < 0.01, *** *p* < 0.005, **** *p* < 0.001, compared to heparin condition. PRP (platelet-rich plasma), PRP-HA (platelet rich plasma combined with hyaluronic acid), PL (platelet lysates) HUVEC (human umbilical vein endothelial cells). Scale bars: 100 µm.

After 3 days of culture with different platelet-derived preparations, there was a higher number of HUVECs in 10% PRP and 10% PRP-HA compared to control (Figure 1A). Control condition and 10% PL were comparable in terms of cell density.

To assess metabolic activity, we incorporated MTT in the cultures after 3 days of treatment with the platelet-derived products (Figure 1B). Under microscopic examination of the FBS-treated cultures (control), formazan granules were found in intracellular organelles. However, when the cells were treated with 10% PRP or 10% PRP-HA, needle-like crystals appeared on the surface of the cells, representing exocytosed MTT formazan, reflecting an increase of cell metabolic activity and exocytosis. PL-treated cells had a mixture of intracellular and exocytosed MTT. In a further experiment, we added Crystal violet as a dye to study the effect of platelet-derived products on cell viability (Figure 1C). After 3 days of treatment, more viable violet cells were found in PRP and PRP-HA-treated cultures. The dye was dissolved out of the cells and the optical density was determined (Figure 1D). PRP and PRP-HA (10–40%) increased cell viability in a dose-dependent manner compared to the control condition. PL also increased cell viability but to a lesser extent.

### 3.2. Platelet-Derived Products Have Different Effects on HUVEC Tube Formation

**Figure 2:**
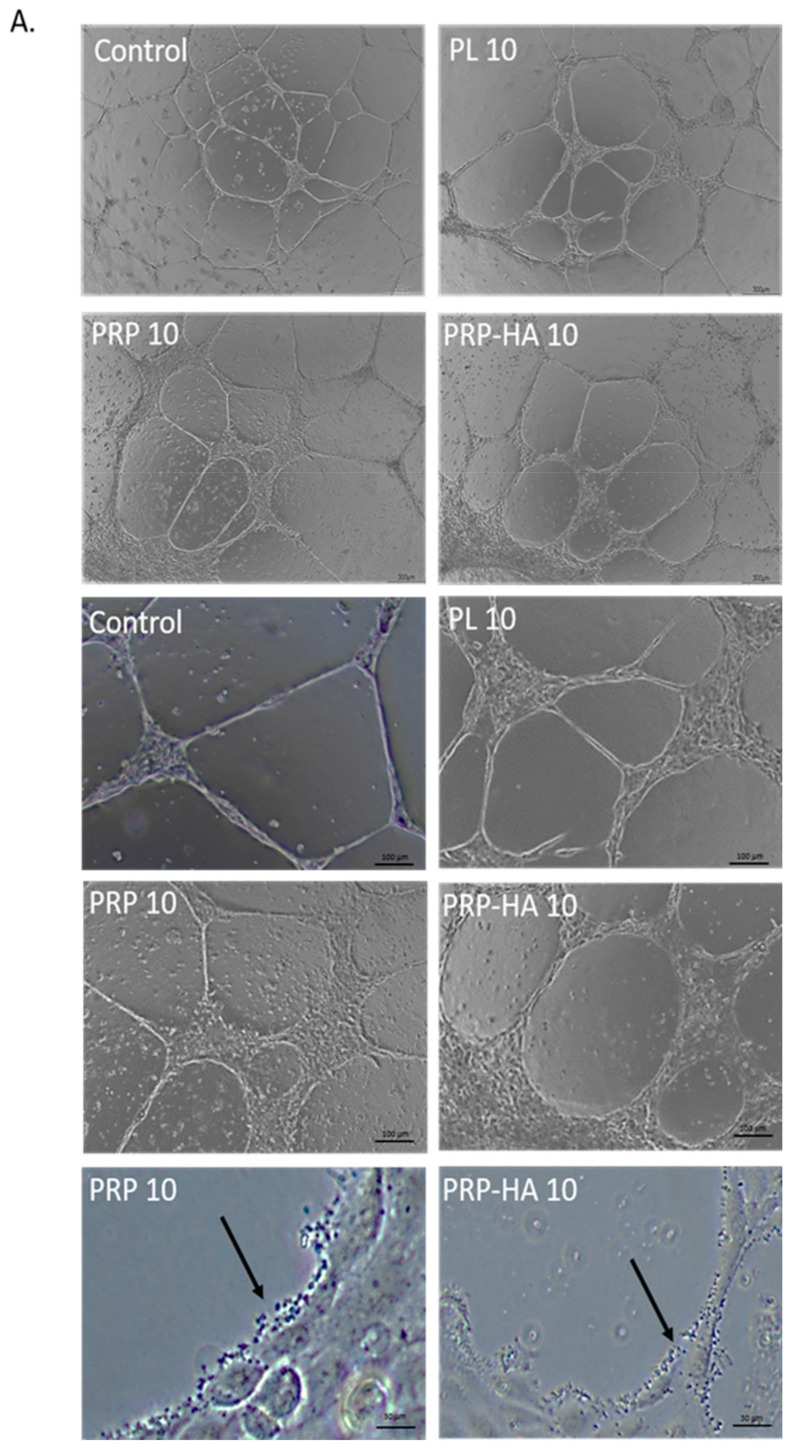

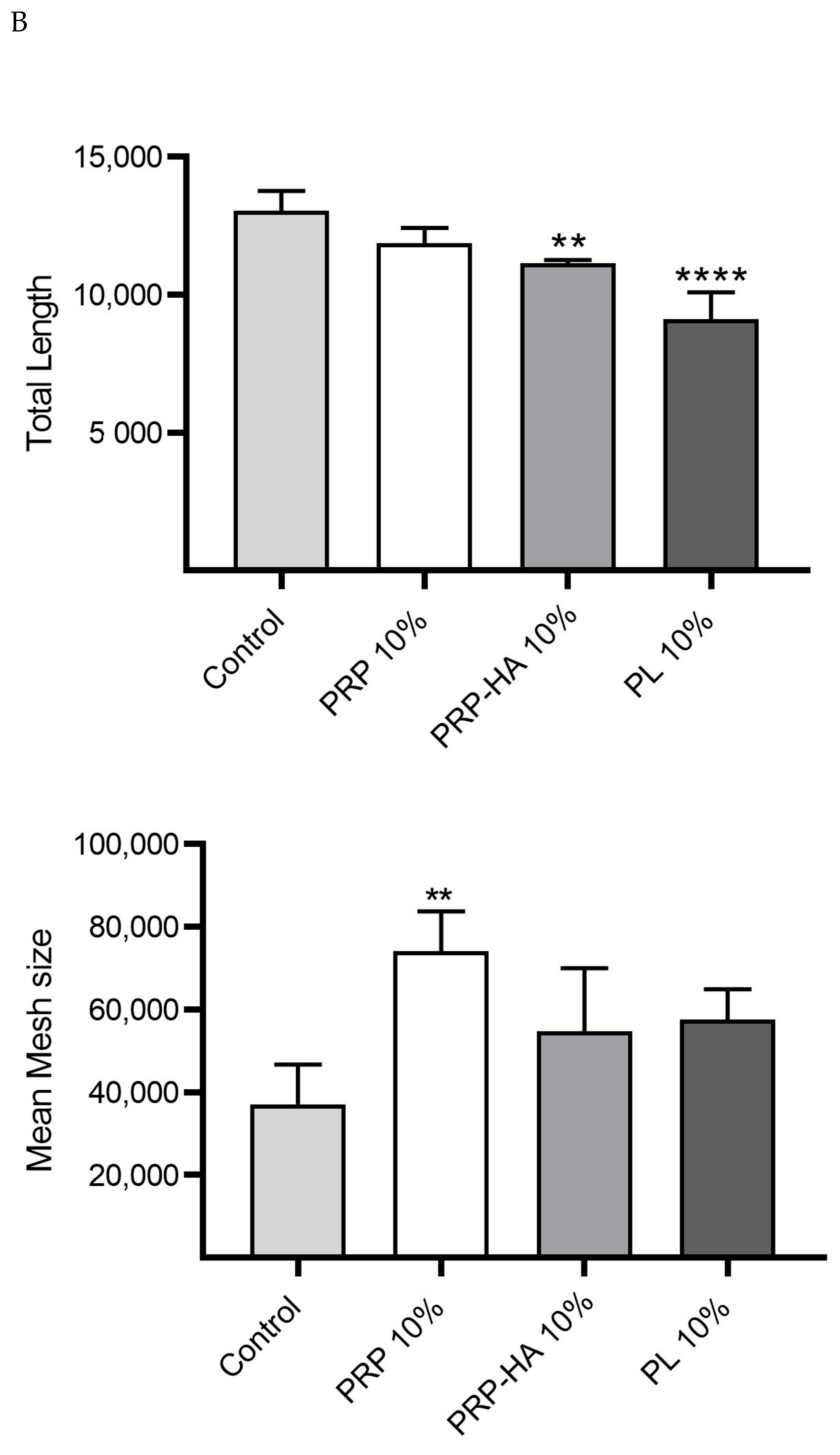
Platelet-derived products have different effects on HUVEC tube formation on Matrigel (**A**). Representative images of the tube formation experiment after 6 h of incubation with control medium (heparin), or platelet-derived products. Scale bars = 300 µm. Arrows indicate platelets attached to the endothelial lining of the pseudo-vascular structure (**B**). Graph shows the average area without cells relative to the total area., ** *p*-value < 0.01, **** *p* value < 0.0001 compared to control cultures.

We used the tube formation assay to investigate the effect of platelet-derived products on endothelial cell (EC) angiogenesis. To decrease any influence by growth factors present within our angiogenesis assay, we used a growth factor reduced Matrigel. As shown in Figure 2A, incubation of HUVEC with non-activated platelet-derived products for 6 h affected the tube formation process. The total length of the network was non -significantly decreased with PRP and significantly decreased with PRP-HA and PL treatments while mean mesh size significantly increased with PRP (Figure 2B). Increased mean mesh size indicates higher tube elongation with PRP 10%. In the zoomed-in images, we observed that platelets were in direct contact with the vascular structures formed on Matrigel surfaces. Endothelial morphogenesis on Matrigel is a model where the network is limited in space and in a short period of time and only reflects a part of the whole angiogenic process. This model is widely used to screen angiogenic compounds and is limited for more complex angiogenic activities evaluation [23].

### 3.3. Platelet-Derived Products Differentially Modulate Angiogenesis in the 3D Fibrin Bead Assay

**Figure 3:**
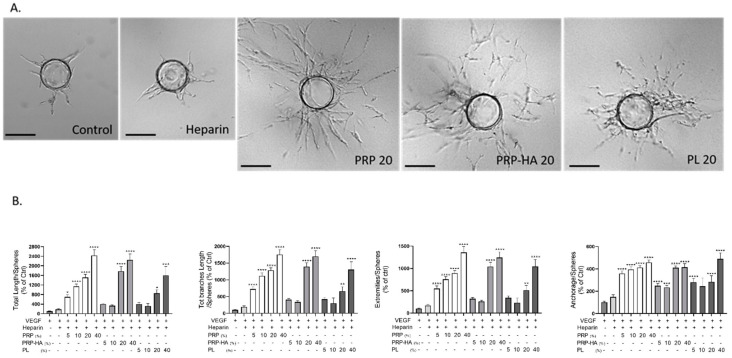
Platelet-derived products differentially modulated angiogenesis in a 3D fibrin gel. (**A**) 3D HUVEC cultures were treated with either PRP, PRP-HA or PL for 4 days. Representative images showing massive enhancement of angiogenesis (20% PRP, 20% PRP-HA) or slight endothelial proliferation from the EC coated beads (20% PL) compared to control conditions (control and heparin) at day 4. Scale bar: 150 µm. (**B**) Quantification of morphometrical parameters of the capillary network was performed by a computerized method on images taken on day 4. Representative parameters measured were total length, total branch length, number of extremities and number of anchorage junctions per sphere. Graphs are representative of three independent experiments. One hundred spheres were quantified for each experimental condition and compared to the heparin-treated cultures. Significant from heparin as measured by the one-way ANOVA followed by Dunett’s multiple comparison test (* *p* < 0.05, ** *p* < 0.01, *** *p* < 0.005, **** *p* < 0.001).

The fibrin bead assay (FBA) is a model that mimics sprouting angiogenesis (Figure 3). The basic steps of sprouting angiogenesis include enzymatic degradation of the capillary basement membrane, endothelial cell proliferation, directed migration of ECs, tubulogenesis (EC tube formation), vessel fusion, vessel pruning and pericyte stabilization.

PRP based preparations and PL elicited different angiogenic responses when tested in the FBA as in EFTA, indicating that they were acting on different steps of the angiogenic process. Thanks to the powerful automatic quantification method we have developed [18], we could assess the changes in the morphometrical parameters of the neovessels formed in the fibrin matrix. Examples are the total length of the microvascular network, the anastomosis in the vessels (branches), the number of capillaries arising from the beads (anchorage) and the number of vessel tips showing the complexity of the network (extremities).

PRP based preparations stimulated all steps of the angiogenic process as massive sprouting of a branched microvessel network was observed by optical microscopy (Figure 3A). PRP and PRP-HA gave better angiogenic response than PL, as the total length of the neovascular network, total branch length, extremities and anchorage were highly stimulated compared to the control condition (VEGF +/− heparin treatment) (Figure 3B).

PRP was the most potent angiogenic preparation, significatively stimulating microvascular network formation from 3 (at 5% PRP) to 12-fold (at 40% PRP). PRP-HA also stimulated angiogenesis but to a lesser extent than PRP. A minimum of 20% PRP-HA was required to elicit the same angiogenic response as 5% PRP. Platelet lysates needed to be highly concentrated (40%) to elicit the same angiogenic response as PRP and PRP-HA.

### 3.4. Profiling of the Angiogenic Protein Secretome of the PRP, PRP-HA, PL Preparations Alone and after Culture with HUVEC

**Figure 4:**
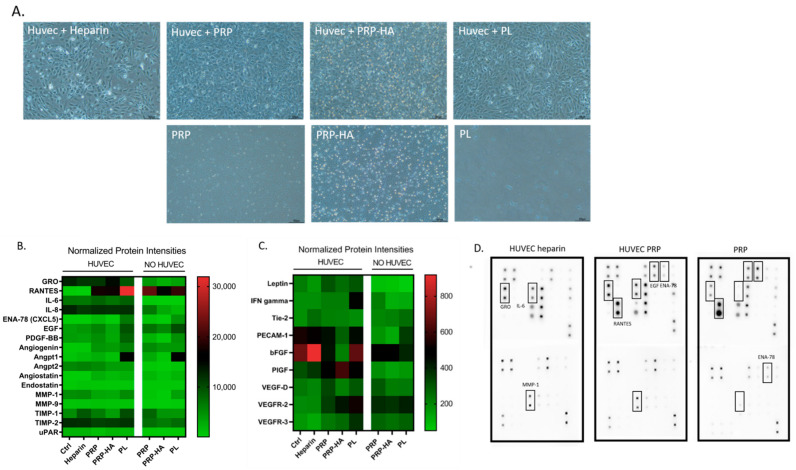
Secretome profiling of angiogenic proteins present in PRP, PRP-HA, and PL preparations and in the medium after culture with HUVEC. (**A**) Representative images of the cultures used to produce conditioned medium for secretome profiling on angiogenesis cytokine arrays. Upper line: HUVEC in the presence of heparin (control medium) or PRP, PRP-HA or PL. Lower line: medium containing PRP, PRP-HA or PL, no cells. Scale bars: 300 µm. (**B**,**C**) Heat map analysis representing the average pixel density of the duplicated spots for each angiogenic-related protein highlighted the array quantified by densitometry. Variation in color intensities represents differential secretion of angiogenic proteins by HUVEC, platelet-derived products (PRP, PRP-HA or PL) or the combination of both. (**D**) Example of the human angiogenesis antibody array composed of duplicated spots of 43 angiogenic-related factors performed with cultures supernatants of HUVEC with heparin, HUVEC with PRP or PRP alone.

To further identify the relevant mediator(s) responsible for the proangiogenic activity, an angiogenic cytokine antibody array was performed. We evaluated growth factor release from the different platelet-derived preparations in the absence of other cells and when added to HUVEC cultures. The array was performed on the proteins released from the three preparations in the presence or absence of HUVEC, cultured for 48 h in these conditions: heparin treatment (control), 10% PRP, 10% PRP-HA, 10% PL (Figure 4A).

Analysis (heat map arrays) of proteins secreted at a high level of secretion is shown in Figure 4B and low level of secretion in Figure 4C. The PRP preparation contained large amounts of GRO, RANTES, IL-8, EGF, Angiogenin, Angiopoietin 1, Angiostatin, TIMP-1, and TIMP-2. In the presence of HUVEC, the amount of GRO, IL-8, TIMP-2 in the culture medium increased and RANTES, EGF, TIMP-1 decreased. There was no change in secretion of the other factors in comparison to PRP alone.

The PRP-HA preparation showed a similar profile to PRP but the amount of secreted factors was lower, both in the PRP-HA preparation and when added to HUVEC cultures. This was expected due to the reduced concentration of PRP in this preparation compared to the PRP alone preparation.

The PL preparation contained large amounts of RANTES, CXCL5, EGF, PDGF-BB, Angiogenin, Angpt-1, MMP-1, TIMP-1 and TIMP-2. When PL were added to the HUVEC cultures, RANTES and IL-8, TIMP-1 and TIMP-2 increased suggesting a synergistic effect with HUVEC.

The analysis of proteins secreted at low levels (low densities) is shown in Figure 4C. The PRP preparation had a low secretion of IFN-gamma, Tie-2, PECAM-1, bFGF, VEGF-D, VEGFR-2 and VEGFR-3. When PRP was added to HUVEC cultures, leptin, PECAM-1, and PlGF increased. PRP-HA cultured samples had a similar profile, except that in the presence of HUVEC, bFGF was not increased and PLGF increased more in comparison to PRP alone. Concerning the PL preparation, the factors secreted at low levels were comparable to those of the other two preparations except for PECAM-1, which was present in a larger amount in the PL preparation. Figure 4D shows examples of three angiogenesis cytokine antibody array membranes for HUVEC cultures with heparin, HUVEC cultured with PRP or PRP alone with no cells. The boxes demonstrate some of the angiogenic cytokines that were identified in the different preparations.

### 3.5. Replicative Senescence Associated Beta-Galactosidase (SA-β-gal) Staining of HUVEC

**Figure 5:**
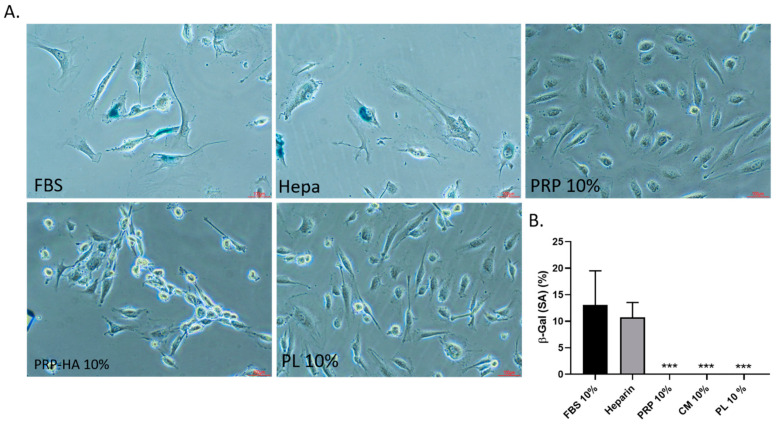
Senescence associated beta-galactosidase (SA-β-gal) staining of HUVECs. (20× objective lenses) at passage 6 treated with FBS, Heparin, 10% PRP, 10% PRP-HA or 10% PL as indicated in the figures. Beta-gal positive cells appear in blue in FBS and heparin culture conditions (**A**). Proportion of SA-β-gal positive HUVECs in A-E culture conditions. *** *p* < 0.005 (**B**).

To evaluate the effect of platelet-derived preparations on HUVEC senescence, we induced aging in vitro by cultivating the cells at a low density [24] from passages 2 to 6 (Figure 5).

With in vitro aging, cells and their nuclei increase in size, and more apoptotic cells appear [22]. In this assay, we tested 10% FBS, Heparin 2UI/mL, 10% PRP, 10% PRP-HA and 10% PL. Long-term serial cultivation in FBS or heparin media induced senescent cells, while no senescent cells were observed with platelet-derived treatments.

## 4. Discussion

Tissue engineering aims to generate large portions of tissue to repair major defects. In this field, we thus need a deeper understanding of the mechanisms and actors regulating the angiogenic process. Angiogenesis is a complex phenomenon where key features are: (i) temporal regulation, (ii) spatial organization of the stimuli, (iii) cellular crosstalk and (iv) active remodeling and interaction with the extracellular matrix (ECM) [25]. In physiological angiogenesis, a variety of growth factors tightly regulate functional blood vessel formation. The main angiogenesis regulators released by platelets are: vascular endothelial growth factor (VEGF), basic fibroblast growth factor (bFGF), and platelet derived growth factor (PDGF) [7].

The purpose of the current study was to better characterize the angiogenic potential of three different platelet-based preparations: PRP, PRP-HA and PL on different 2D and 3D angiogenic models. We used freshly prepared PRP and PRP-HA and commercial PL. Currently, there is no in vivo evidence to indicate which type of preparation has the best angiogenic activity [4].

In this study, we used two specific medical devices (CuteCell^TM^ and CellularMatrix^TM^) and a standardized method to prepare the PRP-based preparations, allowing us to work with single donors and not pooled plasma as the interindividual variations are very low. In previous studies, we have demonstrated that the PRP prepared by our device contains 1.6 times more platelets than whole blood, with a platelet recovery rate in PRP from whole blood of 96% [16,17].

The use of PRP as a suitable source of autologous growth factors is considered as a promising therapy for tissue regeneration. The definition of PRP in most of the studies is “the component of plasma fraction of autologous venous blood with platelet counts in the range between 4 and 6 times above baseline”, which is the range that is considered to be of therapeutic benefit [26].

This empirical value has never been scientifically validated nor has its superiority to lower concentrated PRPs, prepared with effective technologies, ever been demonstrated in comparative clinical studies.

This level of platelet concentration usually applies to PRPs highly contaminated with red and white blood cells. As these cellular contaminants delay the healing process, a higher concentration of platelets is probably needed in these types of PRPs to compensate for the negative effect of red and white blood cells on the healing process [27].

At the cellular level, we and others have already demonstrated that PRP with a low supra-physiologic concentration of platelets has a better effect on cell proliferation than highly platelet concentrated PRP [16,17,28]. CuteCell^TM^ PRP is considered a leukocyte-poor PRP, containing 1 × 10^3^ leukocytes/mm^3^ of blood. The white blood cell level in this preparation is drastically reduced, with a preferential depletion (96.7%) of the pro- inflammatory granulocytes. The remaining white blood cells are mostly lymphocytes and monocytes. Leukocyte angiogenic activity was evaluated by Giusti et al. 2018 [29] in wound healing and tube formation models. They concluded that the ability of PRP to affect the in vitro endothelial response is not dependent on the presence of leukocytes. On this basis, we assume that any of the angiogenic responses observed in our study are not due to the presence of the very low level of leukocytes present in our PRP-derived preparations.

Previous work on angiogenesis revealed that a dose of 1.5 × 10^9^ platelets/mL is needed for tissue repair mechanisms to induce a functional angiogenic response through endothelial cell activity [30]. However, the authors found that higher concentrations of platelets reduced their angiogenic potential in follicular and perifollicular angiogenesis. Our PRP device allowed us to retrieve the majority of the platelets in a functional state. This concentration, close to physiological level, is obtained by the resuspension of the platelets in the whole volume of plasma. Plasma by itself, contained very important angiogenic mediators like IGF-1 and HGF [31].

PRGF (plasma rich in growth factors) is another PRP-based product in which the PRP is activated prior to use and forms an in situ-generated fibrin-matrix delivery of GF and other active biomolecules. This product has also been shown to stimulate cell proliferation and reduce apoptosis in primary HUVEC and skeletal myoblasts [32].

Concerning the influence of HA, numerous studies have shown that HA signaling plays an important role in the regulation of angiogenesis, mainly by influencing endothelial cell behavior. The molecular weight of the HA is of prime importance as both high molecular (HMWHA) and low molecular weight (LMWHA) are potent regulators of angiogenesis. However, they display opposite effects on EC proliferation and motility. LMWHA are pro-angiogenic while HMWHA are anti-angiogenic. The HA used in our preparation is of medium molecular weight (1550 kDa) and its synergistic effect with PRP on in vitro angiogenesis has never been determined before. As HA will form a hydrogel where the platelets are entrapped, we believe that the spatial organization of the angiogenic stimuli is modified in comparison to PRP alone.

In our study, we first used 2D HUVEC cultures to demonstrate that the three platelet-based preparations could induce proliferation of HUVECs. In a MTT assay, we showed that PRP based preparations enhanced metabolic activity and exocytosis of formazan. The exocytosis of formazan-containing vesicles gives rise to needle-like formazan crystals at the cell surface [33]. By exocytosis, the HUVEC intracellular vesicles will fuse to the plasma membrane and release many other active biomolecules and possibly other angiogenic factors, promoting a synergistic angiogenic effect between PRP based preparations and HUVEC.

The effect of these preparations on endothelial morphogenesis on a Matrigel matrix (in terms of branch length and mesh size) was then assessed using the tube formation assay, a model to study endothelial morphogenesis in capillary-like structures. PRP treatment negatively affected the total length of the network. Nevertheless, its complexity reflecting the tube elongation, was increased. Some groups have already demonstrated the tube formation promoting effect of platelets (5 × 10^5^) alone (plasma removed) [34], or on sonicated and frozen PRP extracts (i.e., PL) [35]. We believe that our tube formation results are related to the dose of PRP and the timing of the release of GFs. The 6 h culture period in this model allow to study the first steps of the angiogenic process. This timing is too short to allow PRP and PRP-HA to release all their angiogenic GFs, as they were not pre-activated; whereas addition of PL introduces a too large a quantity of growth factors at the same time and may have a negative effect on EC morphogenesis. Indeed, Giusti et al. 2009 [30] demonstrated that tube formation was highly dependent on the platelet concentration, with high concentrations inhibiting the cellular effects of the platelet gel-derived supernatant. This assay is traditional assay that evaluate the endothelial cord formation. It is insufficient to mimic the complexity of angiogenesis as these assays are 2D and ECs often form incomplete lumens. Moreover, lumen formation on Matrigel is found with other cell types (prostate carcinoma and glioblastoma) [23].

Apart from dose-dependency, the effects of platelet-based preparations on endothelial cell activity appear to be highly time-dependent. As explained earlier, angiogenesis needs temporal regulation.

In PL, lysis completely degrades the platelets, thereby releasing all platelet-derived growth factors. Consequently, treatment of cells with PL results in an immediate response due to the introduction of a larger number of GFs [36] to the culture medium in comparison to PRP based preparations. However, the growth factors in platelet lysates have a short half-life [37], meaning a rapid depletion in the culture medium after stimulation of the initial step of the angiogenesis process, i.e., endothelial cell proliferation. Therefore, although EC were stimulated and proliferated in our 3D angiogenesis model, they did not form proper vessels.

PRP and PRP-HA are freshly prepared autologous platelet preparations. We chose not to use activation methods such as thrombin activation because the slow and orchestrated release of growth factors is needed for the complete angiogenic process to occur [38]. Moreover, from a technical point of view, activation would result in a 3D fibrin PRP gel, which would not have been suitable for the chosen assays.

PRP and PRP-HA contain live platelets that continuously deliver growth factors and nutrients that stimulate the whole angiogenesis process either directly in EC or indirectly (through a paracrine effect on NHDF present on the top of the fibrin matrix in FBA). Non-activated PRP and PRP-HA will slowly release their GF for up to 10 days [16] and this is expected to lead to an appropriate stimulation of the whole sequence of the angiogenesis steps and formation of a mature capillary network. Thanks to the 3D angiogenesis assay used in this study, we have identified that PRP and PRP-HA have a dose-dependent effect on angiogenesis. A low concentration of PRP (5%) significatively stimulates the all the morphometrical parameters measured, whereas at least 20% of PRP-HA or PL is needed to achieve the same response. The PRP-HA preparation is a less potent angiogenic stimulator in the FBA as the concentration of PRP in this preparation is reduced (60%PRP: 40% HA) compared to the PRP alone preparation.

To investigate which angiogenic factors are secreted in the respective platelet-based preparations in the presence or absence of HUVEC, we used an angiogenesis protein array to determine the differences in the relative protein level secretion of the different angiogenic factors.

Among the 26 cytokines, GRO, RANTES, IL-6, IL-8, EGF, ENA-78 showed the highest secretion modulation between the platelet derived preparations alone or with the presence of HUVEC. GRO, ENA-78 and IL-8 are high affinity CXCR-2 (IL-8 receptor beta) ligands [39] that present pro-angiogenic effects via the CXCR-2 signaling pathway acting on endothelial cell chemotaxis [40]. RANTES was present in high amounts in all three preparations and when cultured with HUVEC cultures as well, with a maximal in response with PL treatment. RANTES or CCL5 is a chemokine that has a role in wound repair and is mainly released by platelet and smooth muscle cells, and to a lesser extent by endothelial cells [41]. The pro-angiogenic effect of RANTES has been demonstrated in an ischemia-induced model of angiogenesis [42]. Interestingly, IL-6 is produced by HUVEC independently of the presence of the platelet-derived preparations and is a direct inducer of angiogenesis [43].

One of the most important growth and survival factor for endothelium is vascular endothelial growth factor (VEGF), a potent inducer of angiogenesis and endothelial cell proliferation [44]. In our angiogenic array, there was no detectable VEGF in any of the conditions. We believe that during cultivation with platelet derived preparations, HUVEC adsorbed the free VEGF released from the preparations into the supernatants by VEGF binding to its receptor, VEGF receptor 2 (VEGFR-2). Indeed, in HUVEC cultured in the presence of the three preparations, there was an increase of VEGFR-2. Thanks to this potent angiogenic stimulus, they will become activated and achieved microvessel-like structure formation.

Earlier in this study, we demonstrated that PRP-derived preparations increased HUVEC exocytosis. In endothelial cells, Weibel-Palade bodies are secretory organelles that release peptides such as von Willebrand Factor, Angpt2 and IL-8 by exocytosis [45]. In HUVEC stimulated with PRP derived preparations, we detected Angpt2 and Il-8 secretion. This synergistic effect is achieved with both the presence of HUVEC and living platelets. PRP based preparations secreted lower amounts of Angpt2 and Il-8 when cultured alone. Interestingly, we found a high release of bFGF in the HUVEC cultured in the presence of heparin. Heparin was shown to interact with bioactive bFGF, preventing its rapid degradation [46].

Other angiogenic factors were mostly detected in PL: Angpt1, PECAM-1, MMP-1. Those large amount of GF, delivered at the same time, may act as potent stimulants of endothelial motility and proliferation. In a non-orchestrated manner, no proper angiogenesis is achieved. This was observed when PL were tested in the fibrin bead assay. Increased expression of ENA-78, RANTES, IL-8 and GRO without the presence of HUVEC were also found in Leukocyte- and Platelet-Rich Fibrin (L-PRF) conditioned medium [47]. The differences in the amount of secreted factors between PRP and PRP-HA can be attributed to the lower platelet concentration in the PRP-HA compared to PRP, and to the entrapment of growth factors in the HA hydrogel.

Recent clinical work is emphasizing the effect of PRP on skin rejuvenation [48,49]. Indeed, with aging, cutaneous blood vessels undergo pronounced alterations [50]. In order to evaluate the rejuvenation potential of the platelet-derived products, we induced endothelial aging in vitro. All three preparations were able to prevent HUVEC from entering senescence with a possible longer angiogenic potential.

## 5. Conclusions

PRP, PRP-HA and PL elicit different angiogenic responses. We demonstrated that even that the three preparations are platelet-derived, they contain diverse mixtures of growth factors that differently triggers the steps of the angiogenic process spatially and timely.

The PL are potent stimulators of endothelial biological activities but in a non-orchestrated manner that impairs proper endothelial tube formation. PRP and PRP-HA containing living platelets elicit the best angiogenic response in the complex 3D FBA model that mimics the whole angiogenic process. PRP and PRP-HA may also present anti-aging properties on HUVEC as endothelial senescence is decreased. We believe that HA, as a natural biodegradable hydrogel is promoting a controlled release of PRP growth factors. The secretome profiling explain the biological activities through differential potent angiogenic factors secretion when the platelets are cultured alone or in the presence of HUVEC.

In conclusion, PRP alone or combined with HA are growth factors reservoirs that promote angiogenesis for in vitro cell tissue-engineering applications. Moreover, the results are of great interest for clinical applications where platelet-derived preparations are used for a in situ angio-regenerative therapy to speed up the wound healing and tissue repair.

## Data Availability

The data presented in this study are available on request from the corresponding author.

## Abbreviations

PRP	platelet-rich plasma
PRP-HA	platelet-rich plasma combined with hyaluronic acid
PL	platelet lysates
2D	two dimensional
3D	three dimensional
HUVEC	human umbilical vein endothelial cells
EC	endothelial cells
NHDF	normal human dermal fibroblasts
RBC	red blood cells
MPV	mean platelet volume
FBS	FETAL BOVINE SERUM
PBS	phosphate buffered saline
MTT	3-(4,5-dimethylthiazol-2-yl)-2,5-diphenyltetrazolium bromide
GRO	growth-regulated oncogene
RANTES	Regulated upon Activation, Normal T Cell Expressed and Presumably Secreted
IL-6	interleukin-6
IL-8	interleukin-8
EGF	epidermal growth factor
ENA-78	epithelial-neutrophil activating peptide-78
CXCR-2	C-X-C Motif Chemokine Receptor-2
ANGPT1	angiopoitein 1
ANGPT2	angiopoitein 2
PECAM-1	platelet endothelial cell adhesion molecule-1
MMP-1	matrix metalloproteinase -1
VEGF	vascular endothelial growth factor
PDGF	platelet derived growth factor
BFGF	basic fibroblast growth factor
